# Severe intraocular lens opacification after scleral suturing in a patient with retinitis pigmentosa

**Published:** 2019

**Authors:** Piotr Kanclerz, Andrzej Grzybowski

**Affiliations:** * Private Practice, Gdańsk, Poland; ** Department of Ophthalmology, University of Warmia and Mazury, Olsztyn, Poland; *** Institute for Research in Ophthalmology, Foundation for Ophthalmology Development, Poznan, Poland

**Keywords:** intraocular lens, opacification, phacoemulsification cataract surgery, retinitis pigmentosa, scleral suturing, vitrectomy

## Abstract

**Objective:** To report a case of late intraocular lens (IOL) opacification after scleral suturing in a patient with retinitis pigmentosa.

**Methods:** A 36-year-old man presented with visual impairment increasing gradually over the preceding three months. Seven years earlier, he underwent bilateral phacoemulsification cataract surgery with implantation of a Rayner 620H IOL in the left eye, and a Quatrix P12 IOL in the right eye. Five months prior to admission, scleral suturing of the subluxated IOL in the left eye was performed due to subluxation. Slit lamp examination revealed significant opacities within the IOL and marked vitreous floaters in the left eye. The previous diagnosis of retinitis pigmentosa and rotary nystagmus were confirmed. Subsequently, he underwent 25-gauge vitrectomy with IOL removal in his left eye. Light microscopy of the explanted IOL revealed unevenly distributed microvacuoles. Subsequently, vitrectomy with IOL removal was performed after 3 months in the right eye.

**Results:** The patient had a good recovery of vision, with a visual acuity of 20/200 in both eyes with contact lenses.

**Conclusions:** The presented case demonstrated a significant increase in IOL opacification following scleral suturing. The presumed pathogenesis includes IOL microdeformation with development of IOL matrix cavities and intense inflammatory response related to scleral suturing.

## Introduction

Postoperative opacification of the intraocular lens (IOL) optic after uneventful cataract surgery is infrequent. Usually, development of IOL opacities is a slow progressive process, which is found several years after primary implantation. In recent years, as the anticipated duration of IOL in the eye has significantly increased, the physicochemical properties and IOL endurance allowing the lens to keep its optical properties gained importance. In some cases, IOL opacification might result in vision impairment and require IOL exchange or explantation.

This study presents an uncommon case of significant visual impairment associated with IOL opacification.

## Case report

**Fig. 1:**
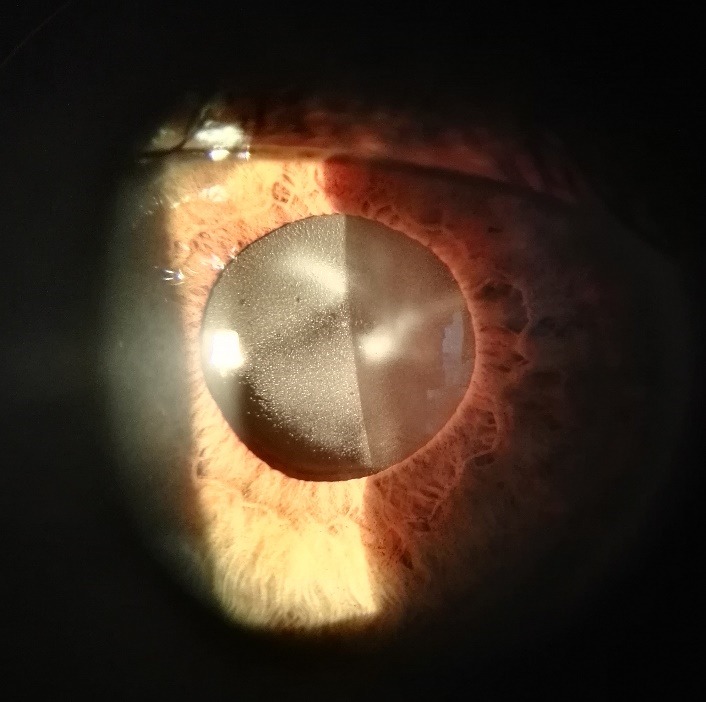
Preoperative slit lamp examination of the left eye manifesting severe IOL opacification

A 36-year-old man presented with a significant visual impairment that had increased gradually over the preceding three months. The visual acuity in his right and left eyes was only hand movement. Seven years earlier, he underwent phacoemulsification cataract surgery in both eyes, with a Rayner 620H hydrophilic acrylic IOL (Rayner Intraocular Lenses GmbH, Bamberg, Germany) implanted in the left eye, and the right eye received a Quatrix P12 hydrophilic acrylic IOL (Bausch+Lomb Inc., Rochester, NY, USA). Five months prior to the admission, scleral suturing of the subluxated IOL in the left eye was performed. Slit lamp examination revealed significant focal opacities within the IOL and marked vitreous floaters in the left eye (**Fig. 1**). The right eye presented capsular phimosis and iridodonesis. The previous diagnosis of retinitis pigmentosa, myopia, and rotary nystagmus were confirmed. The patient did not suffer from glaucoma or diabetes, nor received any continuous topical treatment.

Subsequently, he underwent 25-gauge vitrectomy in his left eye with intraocular IOL fragmentation and removal through a 3.0 mm clear corneal incision. Due to high axial length and unclear reason for IOL opacities development, a new IOL was not implanted. Because of anisometropia, intense anterior phimosis, and increased mobility of the bag-IOL complex in the right eye, vitrectomy with IOL removal was performed after 3 months. Postoperatively, the right eye and left eye had a visual acuity of 20/ 200 with hyperopic correction of +5.5 DS and +6.0 DS, respectively. The patient currently wears contact lenses and is satisfied with the visual outcome, as he can walk unassisted.

**Fig. 2:**
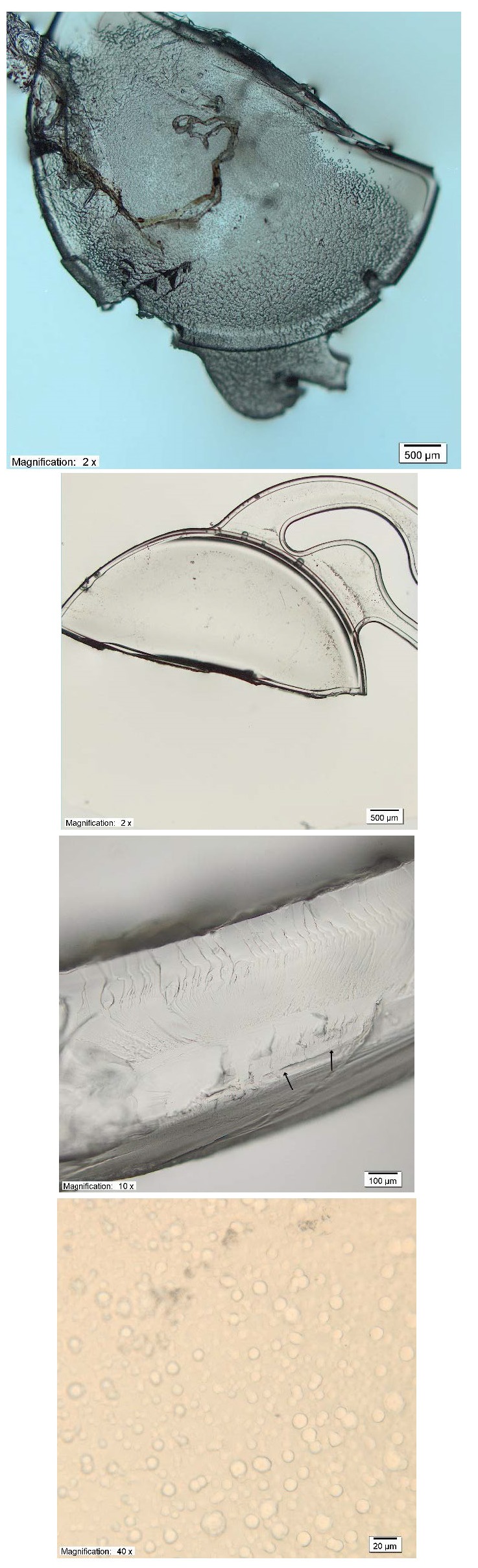
Light microscopy of the removed IOL. Clockwise from upper left: severe opacifications seen on the optic plate and IOL haptic ; opacifications are located unevenly and are not present in the other parts of the IOL; several microvacuoles sized from 5μm to 22 μm; cross-section of the explanted IOL revealed vacuoles were present (indicated by arrows) predominantly in the IOL surface and subsurface

Light microscopy of the explanted dry IOL revealed unevenly distributed microvacuoles, present partially on the optic plate and on one haptic (**Fig. 2**). Higher magnification showed that the microvacuoles range from 5–22 μm in size, and are distributed mainly in the unevenly under the surface of the IOL.

## Discussion

The reported types of late postoperative IOL opacifications are: calcification, glistening, subsurface nanoglistenings and snowflake degeneration [**1**]. Calcification presents mainly in hydrophilic IOLs as deposits of calcium on the IOL surface. Calcification is divided into primary, secondary, and false positive calcification [**2**]. Secondary IOL calcification is related to environmental circumstances including preexisting diseases or breakdown of the blood-aqueous barrier. Glistenings are small (1.0 to 20.0 μm) microvacuoles that develop in the IOL optic when it is placed in an aqueous environment [**3**]. They were reported in all IOL types, however, most commonly are observed in hydrophobic acrylic IOLs. Glistenings are believed to develop for up to 90 days postoperatively, however, an increase in glistenings was reported 10 years [**4**] and 15 years [**5**] after primary implantation, with no indication that the phenomenon will level off.

In the presented case, severe increase in opacification of hydrophilic IOL developed seven years after implantation, and two months after scleral suturing. In our case, light microscopy revealed several microvacuoles having a size of typical glistening formation and placed in the superficial IOL layers and not only on the surface. Calcification deposits usually present as clusters or nanocrystallites (500 to 600 μm) or dense formations (10 to 70 μm) situated on the IOL surface [**6**]. However, calcification manifesting as granules that might resemble microvacuoles and distributed in a line parallel to the external optic was also reported [**7**]. Moreover, in vitro studies demonstrated that glistening completely disappears when the IOL is allowed to dry for at least 30 minutes [**8**].

Both glistening and calcification might be related to excessive postoperative inflammation [**2**,**9**]. In the presented case, the intense inflammatory response related to scleral fixation and resulting in marked vitreous opacities potentially extricated symptom development. With that, mechanical manipulations on the IOL during scleral suturing could result in IOL microdeformations, development of matrix cavities, and subsequent accumulation of calcified deposits or aqueous microvacuoles. This could explain why opacifications were found only in certain regions of the IOL. A limitation of the current study was that scanning electron microscopy was not performed. Moreover, the cut sections of the optics were not stained with 1% alizarin red and the von Kossa method, which are used to dye calcium deposits [**10**].

In the presented case, a significant increase in IOL opacification was found seven years after implantation, and was related to scleral suturing of the subluxated IOL. The presumed pathogenesis includes microdeformation of the IOL with accumulation of deposits in the IOL matrix, accelerated by inflammatory response related to scleral suturing.

**Acknowledgements**

The authors would like to thank Dr. Rafał Pęksa from the Department of Pathomorphology, Medical University of Gdańsk for high-quality photos of the intraocular lens.

Dr. Kanclerz reports non-financial support from Visim and Optopol Technology. Dr. Grzybowski reports grants, personal fees and non-financial support from Bayer, non-financial support from Novartis, non-financial support from Alcon, non-financial support from Thea, personal fees and non-financial support from Valeant, non-financial support from Santen, outside the submitted work.
